# Aldosterone, SGK1, and ion channels in the kidney

**DOI:** 10.1042/CS20171525

**Published:** 2018-01-19

**Authors:** William C. Valinsky, Rhian M. Touyz, Alvin Shrier

**Affiliations:** 1 Department of Physiology, McGill University, 3649 Promenade Sir William Osler, Montreal, Quebec H3G 0B1, Canada; 2 Institute of Cardiovascular and Medical Sciences, University of Glasgow, BHF GCRC, 126 University Place, Glasgow G12 8TA, U.K.

**Keywords:** Aldosterone, ENaC, Hypertension, Ion Channels, SGK1, TRPM7

## Abstract

Hyperaldosteronism, a common cause of hypertension, is strongly connected to Na^+^, K^+^, and Mg^2+^ dysregulation. Owing to its steroidal structure, aldosterone is an active transcriptional modifier when bound to the mineralocorticoid receptor (MR) in cells expressing the enzyme 11β-hydroxysteroid dehydrogenase 2, such as those comprising the aldosterone-sensitive distal nephron (ASDN). One such up-regulated protein, the ubiquitous serum and glucocorticoid regulated kinase 1 (SGK1), has the capacity to modulate the surface expression and function of many classes of renal ion channels, including those that transport Na^+^ (ENaC), K^+^ (ROMK/BK), Ca^2+^ (TRPV4/5/6), Mg^2+^ (TRPM7/6), and Cl^−^ (ClC-K, CFTR). Here, we discuss the mechanisms by which ASDN expressed channels are up-regulated by SGK1, while highlighting newly discovered pathways connecting aldosterone to nonselective cation channels that are permeable to Mg^2+^ (TRPM7) or Ca^2+^ (TRPV4).

## Introduction

In 2017, hypertensive blood pressure thresholds were lowered such that stage 1 hypertension commences at 130 mmHg (systolic) and/or 80 mmHg (diastolic) [1]; down from 140 mmHg/90 mmHg [2]. Prior to these changes, global data showed hypertensive rates of 22–30% in the total population [2–6], however with the more stringent definitions, these rates will no doubt climb. Moreover, the prevalence of hypertension is expected to further increase over time due to increasing rates of obesity and a progressively aging demographic [3].

Clinically, hyperaldosteronism is often observed in resistant hypertension [4] and is a common cause of secondary hypertension [5–8]. This is of major significance because hyperaldosteronism is associated with a plethora of cardiovascular comorbidities and is hallmarked by electrolyte dysregulation [9]. Moreover, drugs that target aldosterone and its mineralocorticoid receptor, such as spironolactone and eplerenone, are increasingly being used in the management of various pathologies, including hypertension, heart failure, arrhythmias and renal disease [10,11]. Therefore, it is critically important that the ion regulatory pathways of aldosterone are fully understood to understand the unintended consequences of aldosterone-related treatments.

Ion transport abnormalities in hyperaldosteronism are to be expected, as the earliest research into aldosterone showed that the steroid hormone decreases the excretion of Na^+^ [12] and increases the excretion of K^+^ and H^+^ [13]. Mechanistically, most effects of aldosterone are exerted through the mineralocorticoid receptor (MR), to which aldosterone binds [14]. However, the MR has equal affinity for aldosterone and glucocorticoids [15], a surprising observation since glucocorticoid plasma concentrations are 100–1000 times higher than aldosterone concentrations [16]. To maintain aldosterone sensitivity, aldosterone-sensitive cells express 11β-hydroxysteroid dehydrogenase 2 [17], which converts cortisol to cortisone [18], preventing cortisol from interacting with the MR [17]. Within the kidney, immunohistochemical and immunocytochemical experiments have shown that 11β-hydroxysteroid dehydrogenase localizes to three consecutive segments: the distal convoluted tubule (DCT), connecting tubule (CNT), and cortical collecting duct (CCD) [19,20]. In some species, where the DCT has been subdivided into the DCT1 and DCT2 based on protein expression [21,22], the aldosterone-sensitive distal nephron (ASDN) would commence in DCT2 [19].

### Aldosterone and genomic signaling

The discovery of the high affinity aldosterone receptor, the MR [14], and 11β-hydroxysteroid dehydrogenase in renal (distal tubular) cells [17,19,20,23] opened the possibility that aldosterone-MR signaling may affect ion transporters, of which Na^+^ transporters were the first to be studied. In the kidney, aldosterone increases the transcription of the basolateral Na^+^/K^+^-ATPase [24] and the apical epithelial Na^+^ channel (ENaC) [25]. Synthesis of channels and pumps were classified as late effects since they were only detected after 20 h of 1 μM aldosterone exposure [26,27]. Short-term mechanisms have also been identified, as increases in Na^+^ transport were observed as early as 2.5 h after aldosterone application in cell-based studies. For apical ENaC, 1.5 μM aldosterone increased channel open time, subsequently increasing Na^+^ transport in A6 (amphibian) kidney cells [28]. For the basolateral Na^+^/K^+^-ATPase, 1 μM aldosterone increased the activity of the Na^+^/K^+^-ATPase at physiological [Na^+^]_i_ [26]. Surprisingly, this response was dependent on protein synthesis since cycloheximide, an inhibitor of protein translation [29], blocked the effect [26]. It was speculated that the MR may transcriptionally up-regulate activators and repressors capable of short-term effects on aldosterone targets.

A83, the A6 (amphibian renal cell) equivalent of serum and glucocorticoid regulated kinase 1 (SGK1), was discovered as an aldosterone responsive protein, since 100 nM aldosterone increased A83 mRNA and protein expression. In addition, SGK1 mRNA significantly increased in the distal cortical nephron of aldosterone treated rats (50 μg/100 g), implicating its role in mammalian function. Furthermore, when SGK1 was coexpressed with ENaC in Xenopus oocytes, macroscopic current increased 7-fold [30]. Since this pioneering study, researchers have connected aldosterone-stimulated SGK1 to many ion channels, including those expressed in the ASDN. Therefore, the purpose of this review is to provide a comprehensive overview of the mechanisms by which aldosterone-MR-SGK1 affect ion channel abundance and/or function, while discussing the present limitations of the literature.

### Na^+^ channels

**Figure 1:**
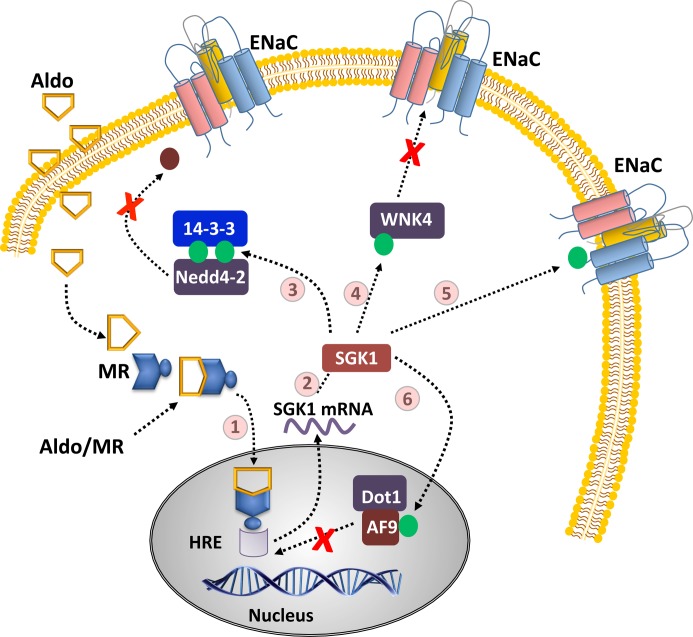
Schematic of aldosterone, SGK1, and ENaC interactions Aldosterone freely crosses phospholipid membranes and binds to the cytosolic mineralocorticoid receptor (MR) (1). The aldo/MR complex translocates to the nucleus, binds to specialized hormone response elements (HREs), and promotes the transcription of aldosterone-regulated genes, including SGK1, which is translated into protein (2). Newly synthesized SGK1 up-regulates ENaC activity through several distinct pathways that reduce ENaC ubiquitination through bi-phosphorylation of Nedd4-2 (3), prevent ENaC endocytosis by phosphorylation of WNK4 (4), recruit silent ENaC channels to active ones by direct phosphorylation (5), and inhibit the transcriptional repressor complex Dot1a–AF9 via phosphorylation of AF9 (6).

There are many regulatory mechanisms whereby SGK1 increases the function of ENaC (Figure 1). First, SGK1 phosphorylates Ser^444^ and Ser^338^ of the E3 ubiquitin ligase ‘Neural precursor cell-expressed developmentally down-regulated protein’ (Nedd) 4-2, which reduces the affinity of Nedd4-2 for ENaC [31,32], and increases the affinity of Nedd4-2 for 14-3-3 [33]. When not phosphorylated, Nedd4-2 interacts with the proline-rich segments of ENaC, causing channel ubiquitination and subsequent internalization from the plasma membrane [34]. By diminishing the Nedd4-2/ENaC interaction and promoting the Nedd4-2/14-3-3 interaction, SGK1 indirectly decreases ENaC internalization, and thus increases ENaC expression at the plasma membrane (Figure 1; pathway 3).

Second, SGK1 phosphorylates ‘kinase with no lysine’ (WNK)4 at Ser^1169^, removing the inhibitory action of WNK4 on ENaC (Figure 1; pathway 4) [35]. Patch clamp studies of the WNK4/ENaC mechanism further showed that WNK4 reduces ENaC current by 50% [36]. Surprisingly, it was observed that the C-terminus of ENaC must be present for the modulation to occur, leading to speculation that Nedd4-2 is involved in the cascade. However, more recent research has indicated that WNK4 decreases the surface expression of ENaC in a Nedd4-2 independent manner, as the C-terminal proline rich motifs of ENaC are not required for WNK4 inhibition [37].

Third, SGK1 is suggested to directly phosphorylate α-ENaC, increasing ENaC electrophysiological function by 2- to 3-fold (Figure 1; pathway 5). However, this response did not affect open channel probability, and since experiments were performed in outside-out macropatches, the authors hypothesized it was due to the conversion of silent channels into active channels. In addition, mutation of Ser^621^ at the C-terminus of α-ENaC abolished the SGK1 effect [38], which is further interesting because Ser^621^ represents the terminal amino acid of the SGK consensus sequence [38,39]. Thus, SGK1 may have a direct regulatory site on α-ENaC.

Fourth, SGK1 may directly increase the transcription of αENaC by disrupting the transcriptional repressor protein complex histone H3 Lys70 methyltransferase ‘disruptor of telomeric silencing alternative splice variant a’ (Dot1a)–‘ALL1-fused gene from chromosome 9’ (AF9), via phosphorylation of Ser^435^ on AF9 (Figure 1; pathway 6) [40]. However, the authors noted that the Dot1a–AF9 interaction was only impaired, not prevented, by SGK1 phosphorylation and that AF9 still bound to the αENaC promoter. Thus, it was concluded that SGK1 may only be a partial component of the mechanism responsible for the inhibition of the Dot1a–AF9 complex.

### K^+^ channels

SGK1 also interacts with the renal outer medullary K^+^ channel (ROMK); an apically located [41,42] K^+^ secretory channel [43] of the distal nephron [44]. Prior to discussing this interaction, it is important to review the nomenclature of the ROMK proteins. ROMK is a three-member splice variant family, where differences between splice variants occur at the mRNA 5′-coding and 3′-noncoding regions [44]. With regard to the 5′-coding region (the N-terminus), ROMK1 contains two predicted targets of PKC phosphorylation (Ser^4^ and Thr^17^), ROMK2 is a truncated protein that lacks both of these sites, and ROMK3 has an extended N-terminus with a PKC-targeting threonine residue, but no equivalent serine residue [44]. These structural differences alter ROMK regulation, as ROMK1 current was inhibited by PKC through phosphorylation of Ser^4^, whereas the activities of ROMK2 and ROMK3 were unaffected [45]. There are also differences in the expression of each splice variant, however all three are expressed in the rat ASDN. Specifically, the DCT expresses ROMK2/3, the CNT expresses ROMK2, and the CCD expresses ROMK1/2 [44].

**Figure 2:**
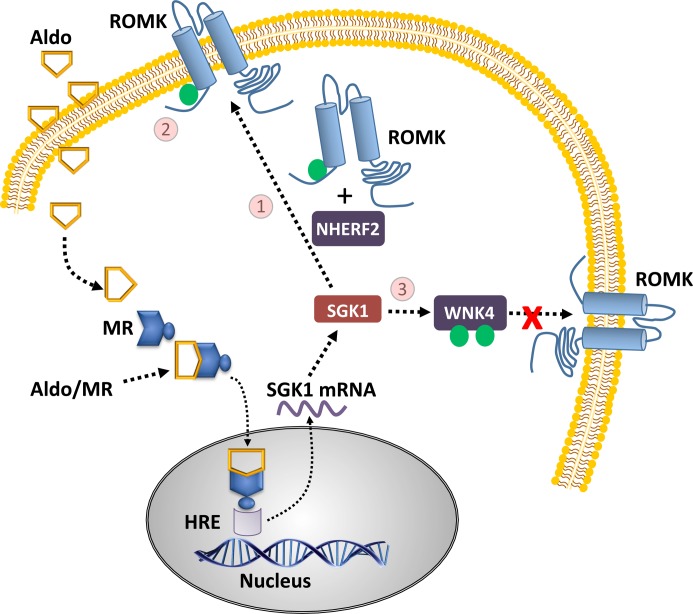
Schematic of aldosterone, SGK1, and ROMK interactions Following an identical cellular entry and SGK1 synthetic pathway discussed for ENaC (Figure 1), aldosterone (through SGK1) up-regulates ROMK activity through three distinct pathways: increased NHERF2-dependent ROMK trafficking via direct phosphorylation of ROMK (1), increased channel function by direct phosphorylation of the same ROMK site (2), and decreased ROMK endocytosis via bi-phosphorylation of WNK4 (3).

In cell-based experiments using exogenous ROMK1 or ROMK2, SGK1 altered ROMK function/expression through three distinct mechanisms (Figure 2). First, SGK1 phosphorylated ROMK1 at Ser^44^, and this was correlated with increased plasma membrane abundance of ROMK1 [46], an effect further dependent on the trafficking/transport protein Na^+^/H^+^ exchange regulatory factor 2 (NHERF2) [47]. These findings indicate that SGK1 increases ROMK1 trafficking, resulting in increased plasma membrane expression (Figure 2; pathway 1). Second, Ser^44^ phosphorylation shifts the pH sensitivity/activation of ROMK1 to more acidic values, increasing electrophysiological function at cytosolic pH 6.6–7.3 (Figure 2; pathway 2) [48]. Third, phosphorylation of Ser^1169^ [35] and Ser^1196^ [49] on WNK4 by SGK1 prevents clathrin-dependent endocytosis of ROMK2 (via the C-terminal NPXY-like motif), increasing the plasma membrane expression of ROMK2 (Figure 2; pathway 3) [50]. Importantly, as Ser^44^ and the C-terminus of ROMK are downstream to the reported N-terminal differences between ROMK1-3 [44], these conclusions may apply to all ROMK splice variants, however this awaits confirmation.

The large conductance Ca^2+^-activated K^+^ channel (BK), also termed Maxi-K^+^, is a K^+^ secretory channel expressed throughout the ASDN [51–56]. BK is primarily stimulated by flow [57] and high K^+^ diets [58–60], although stimulation of BK by membrane stretch has also been reported [61]. An initial study by Estilo et al. [60] suggested aldosterone did not regulate BK in the rabbit CCD. However, it was concurrently reported that aldosterone increased BK mRNA, luminal expression, and K^+^ secretion in the mouse colon [62]. An important difference between these studies was their method of aldosterone stimulation. The CCD study used low Na^+^ diets, whereas the colonic study used high K^+^ diets. Subsequently, in a mouse study where aldosterone was stimulated by high K^+^ diets, it was determined that MR blockade could severely blunt BK expression [63]. A follow-up study by this same group revealed that even with a low Na^+^ and high K^+^ diet, adrenalectamized mice with low aldosterone supplementation had lower apical and total BK expression than control, confirming the necessity of aldosterone for BK up-regulation [64].

The effects of SGK1 on BK function are only beginning to be examined. In a 2017 study comparing control and SGK1 knockout mice, BK whole-cell currents were unaffected, even when animals were fed high K^+^ diets [65]. In addition, ROMK whole-cell currents, amiloride-sensitive whole-cell currents, and amiloride-sensitive Na^+^ excretion were also unaffected in SGK1 knockout mice fed with high K^+^ diets. The latter two results were surprising, as ENaC surface expression was decreased when animals were subjected to similar treatments [65]. To date, there have yet to be any studies that have examined the direct effect of SGK1 on BK plasma membrane expression.

### Ca^2+^ channels

Ca^2+^ reabsorption in the ASDN occurs in part via the epithelial Ca^2+^ channel transient receptor potential vanilloid (TRPV)5 [66–68] and its homolog TRPV6 [68,69]. TRPV5, the first to be studied, was discovered as an apical channel located in the rabbit DCT, CNT, and CCD [66]. For species which subdivide the DCT into DCT1 and DCT2, TRPV5 expression commences in DCT2 [69]. Pertaining to SGK1, coexpression of SGK1, NHERF2, and TRPV5 dramatically increased current in Xenopus oocytes. This change was accompanied by an increase in the TRPV5 surface chemiluminescence, suggesting that SGK1, along with NHERF2, increases the surface expression of TRPV5 [70,71]. The surface expression and function of TRPV6 was also increased when TRPV6 and SGK1 were coexpressed in Xenopus oocytes. This effect did not require NHERF2 [72], differentiating the response from SGK1/TRPV5 [70,71].

TRPV4 is a nonselective cation channel [73,74] expressed on apical membranes of the CNT and CCD [75]. Of relevance to the tubule, TRPV4 is activated by changes in osmolarity [76–78], sheer stress [78–81], and pressure [82]. Indeed, high flow rates over the mouse luminal collecting duct increased [Ca^2+^]_i_, which was abolished in TRPV4 knockout animals [75]. This capacity to increase [Ca^2+^]_i_ has connected TRPV4 to the Ca^2+^-activated BK channel, as TRPV4 potentiators increased flow-dependent K^+^ secretion in wildtype animals whereas urinary K^+^ excretion was significantly decreased in TRPV4 knockout animals [83].

Recently, it has been demonstrated that both aldosterone and high K^+^ diets increase the total expression of TRPV4 in primary and immortalized mouse CCD cells [84]. It was notable that TRPV4 expression in mice treated with MR antagonists was below control, implying that aldosterone constitutively regulates TRPV4 [84]. This study further demonstrated that high K^+^ diets, which should induce aldosterone release [85], increased TRPV4 apical membrane expression and increased flow-mediated [Ca^2+^]_i_ [84]. While SGK1-mediated effects were not explored, the authors noted that prior findings of TRPV4 phosphorylation (at Ser^824^) by SGK1, which increased channel activity, Ca^2+^ influx, and protein stability [86], would explain their aldosterone-mediated effects 84]. Thus, it is possible that aldosterone, through SGK1, increases the expression/function of TRPV4, which increases [Ca^2+^]_i_ in response to sheer stress, and provides the necessary intracellular Ca^2+^ for BK-dependent K^+^ secretion.

### Mg^2+^ channels

The relationship between aldosterone, SGK1, and Mg^2+^ permeable channels represents a largely unexplored field of renal electrolyte regulation. While many Mg^2+^ permeable channels have been identified in DCT primary cells and cell lines, such as transient receptor potential melastatin (TRPM)6 [87–89], TRPM7 [89–91], MagT1 [92,93], and ACDP2/CNNM2 [94], few have been studied with aldosterone. TRPM6 [87,95] and TRPM7 [91,96–98] are further complex, as they comprise Mg^2+^ permeable, nonselective cation channels fused to a C-terminal α-kinase domain. Moreover, the α-kinase domain can be cleaved from both channels and act as a nuclear histone modifier, regulating the expression of thousands of genes [99,100]. Thus, studies examining TRPM6 or TRPM7 must account for the broad-spectrum regulatory capacity of the α-kinase domain.

**Figure 3:**
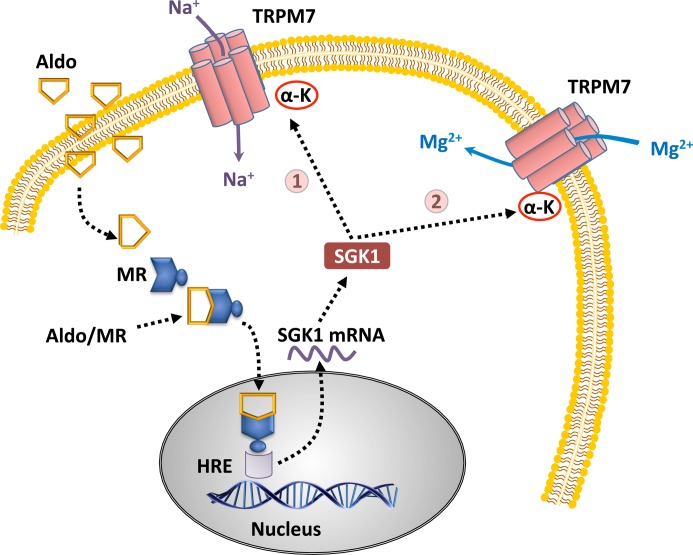
Potential physiological consequences of aldosterone, SGK1, and TRPM7 Aldosterone, through induction of SGK1, increases TRPM7 plasma membrane expression and electrophysiological function via an α-kinase-dependent pathway in expression systems. In the ASDN, where tubular proton concentration is elevated and divalent cation concentrations are low, TRPM7 is likely to function as a Na^+^ channel (1). In tissues where aldosterone is active, extracellular cations are serum-like, and extracellular pH is near 7.4, TRPM7 is likely to function as a Zn^2+^, Mg^2+^, and Ca^2+^ channel (2).

Pertaining to aldosterone, we demonstrated that mice injected with aldosterone have a lower membrane to cytosol fraction of renal TRPM6 compared with control animals, an effect that was rescued when mice were fed high Mg^2+^ diets [101]. We have also studied TRPM7 and aldosterone, including pathways that involve SGK1. In cell-based studies using TRPM7-expressing HEK293 cells, aldosterone increased [Mg^2+^]_i_, ROS, pro-inflammatory mediator expression. Pro-inflammatory mediator expression was only observed in kinase-defective mutants, not wildtype cells [102]. Furthermore, in those same cells, aldosterone increased TRPM7 plasma membrane expression and whole-cell current in an MR and SGK1-dependent mechanism (Figure 3). This effect was abolished in the phosphotransferase inactive K1648R mutant, implying that SGK1 evokes its effects through the α-kinase domain [103].

The consequences of these mechanisms are vast given that TRPM7/6 permeability is governed by electrolytes. In circumstances where extracellular divalent cation concentrations are low and extracellular pH is acidic, such as the distal tubule, TRPM7 and TRPM6 are likely to conduct Na^+^ (Figure 3; pathway 1) [104,105]. However, in extracellular conditions where divalent cation concentrations and pH are serum-like, TRPM7 and TRPM6 are likely to function as nonselective cation channels with Mg^2+^ permeability (Figure 3; pathway 2) [88,106,107]. Further supportive of this rationale, knockout studies targeting TRPM7 or TRPM6 showed that these animals exhibited decreased renal Mg^2+^ excretion and increased fecal Mg^2+^ excretion compared with control [108,109]. While it is tempting to conclude that TRPM7 and TRPM6 function as Na^+^ channels in the ASDN whereas TRPM7 and TRPM6 function as divalent cation (Mg^2+^) channels in the intestine of the KO mice, the loss or reduction of a transcriptionally active α-kinase should severely impact cellular homeostasis. Nonetheless, the dynamic permeability properties of TRPM7 and TRPM6 must be factored into conclusions surrounding their function in aldosterone-sensitive regions.

### Cl^−^ channels

The presence of pathways connecting SGK1 to Cl^−^ transport in the ASDN are less conclusive, however it is highly plausible that aldosterone, through SGK1, is capable of influencing Cl^−^ transport. By a mechanism similar to that described above for ENaC, SGK1 was shown to increase the plasma membrane expression of Cl^−^ permeable ClC-Ka/barttin [110,111] by decreasing the Nedd4-2 interaction with the PY motif of barttin in exogenously expressing Xenopus oocytes [112]. However, in the ASDN, human ClC-Kb/barttin is expressed [113], not ClC-Ka/barttin [114]. Importantly, Nedd4-2 interacts with the barttin subunit [112], and therefore it is possible that SGK1 increases the plasma membrane expression of ClC-Kb/barttin. This hypothesis is further supported by the similarity between ClC-Ka and ClC-Kb (94% sequence homology [115]), although this has yet to be demonstrated.

The mRNA of cystic fibrosis transmembrane conductance regulator (CFTR) has been identified in rabbit DCT [116], and CFTR-like currents have been electrophysiologically recorded in rabbit DCT cells [116,117]. When studied in pancreatic duct adenocarcinoma cells, wildtype CFTR and Nedd4-2 co-immunoprecipitated, implying a physical connection between the two proteins [118]. This interaction was also observed for Nedd4-2 and ΔF508-CFTR, and siRNA knockdown of Nedd4-2 acted as a rescue for ΔF508-CFTR plasma membrane expression. Furthermore, siRNA knockdown of endogenous SGK1 abolished a previously characterized pharmacological rescue of plasma membrane bound ΔF508-CFTR, indicating that SGK1/Nedd4-2 internalization mechanisms mediated the plasma membrane expression of ΔF508-CFTR. Since CFTR is expressed in the aldosterone-sensitive distal nephron, it is also possible that SGK1 modulates CFTR through Nedd4-2 ubiquitination, however this has yet to be determined.

## Conclusions

Aldosterone has long been connected with ion transport and ion channel function. Historically this has emphasized ENaC and ROMK, as Na^+^ and K^+^ dyshomeostasis were some of the first symptoms associated with hyperaldosteronism. Aldosterone signaling cascades, particularly those evoking widely expressed mediators, such as SGK1, have expanded the possible classes of ion channels affected by aldosterone. It is now accepted that aldosterone, through SGK1, has the capacity to modulate ion metabolism through several ion channels, including those that regulate Na^+^, K^+^, Ca^2+^, Mg^2+^, and Cl^−^. Unlike Na^+^ and K^+^ channels, there is a paucity of information regarding aldosterone/SGK1 effects on renal Ca^2+^, Mg^2+^, and Cl^−^ channels. Hence, there is still much to be explored in understanding the mechanistic pathways whereby aldosterone, through its mineralocorticoid receptor and downstream target SGK1, regulate ion channels in the kidney in health and disease. Recognizing that aldosterone influences electrolyte balance beyond its effects on Na^+^ and K^+^ regulation is important because perturbations in renal handling of Mg^2+^, Ca^2+^, Cl^−^, and H^+^ likely influence multiple tissue systems and would impact disease management.

## Abbreviations

ASDN: aldosterone-sensitive distal nephron
BK: large conductance Ca^2+^-activated K^+^ channel
CCD: cortical collecting duct
CFTR: cystic fibrosis transmembrane conductance regulator
CNT: connecting tubule
DCT: distal convoluted tubule
Dot1a–AF9: disruptor of telomeric silencing alternative splice variant a–ALL1-fused gene from chromosome 9
ENaC: epithelial sodium channel
MR: mineralocorticoid receptor
Nedd: neural precursor cell-expressed developmentally down-regulated protein
NHERF2: Na^+^/H^+^ exchange regulatory factor 2
ROMK: renal outer medullary K^+^ channel
SGK1: serum and glucocorticoid regulated kinase 1
TRPM: transient receptor potential melastatin
TRPV: transient receptor potential vanilloid
WNK: kinase with no lysine
